# lncRNA OIP5-AS1 targets ROCK1 to promote cell proliferation and inhibit cell apoptosis through a mechanism involving miR-143-3p in cervical cancer

**DOI:** 10.1590/1414-431X20198883

**Published:** 2020-01-13

**Authors:** Linlin Song, Linlin Wang, Xiaoli Pan, Caihong Yang

**Affiliations:** 1 Department of Gynecology, The General Hospital of Ningxia Medical University, Yinchuan, China; 2 Medical Laboratory Center, The General Hospital of Ningxia Medical University, Yinchuan, China; 3 Department of Pathology, The General Hospital of Ningxia Medical University, Yinchuan, China

**Keywords:** lncRNA, OIP5-AS1, Cervical cancer, miR-143-3p, ROCK1

## Abstract

Opa-interacting protein 5 antisense transcript 1 (OIP5-AS1) is one kind of cytoplasmic long non-coding RNA (lncRNA), which has been demonstrated to play a critical function in multiple cancers. However, the detailed mechanism of OIP5-AS1 in the regulation of cervical cancer progression is still obscure. Here, we demonstrated that lncRNA OIP5-AS1 was upregulated in cervical cancer and was correlated with poor prognosis by bioinformatics studies. OIP5-AS1 depletion inhibited cell proliferation and promoted cell apoptosis in cervical cancer cells. Furthermore, we clarified that ROCK1 was the downstream effector of OIP5-AS1 and OIP5-AS1 acted as a molecular sponge of miR-143-3p. Finally, we verified that OIP5-AS1 exerted its function in the regulation of cervical cancer progression via interacting with miR-143-3p to regulate ROCK1 expression. Our study revealed novel mechanisms about how lncRNA OIP5-AS1 executed its function in cervical cancer and thus provided potential therapeutic targets for the disease.

## Introduction

Cervical cancer (CC) is one of the most common cancer types in women worldwide and a leading cause of tumor-related deaths among women globally (1,2). CC usually arises from the cervix mucosa, which is also regarded as the cervical transformation zone (1,3). There are four main steps for the occurrence and development of CC: virus infection of the metaplasia epithelium in the cervical transformation area, persistence of the virus, development of the continuously infected epithelium into cervical pre-cancer, and infiltration of the epithelial basement membrane. To date, knowledge about the detailed cause and pathogenesis remains vague. Therefore, it is necessary to study the molecular pathways underlying the pathophysiology of CC progression to identify new diagnostic and therapeutic interventions.

Long non-coding RNAs (lncRNAs) are RNAs with a length of more than 200 nucleotides and are not translated into proteins (4–6). Their functional disorders and specific roles in various diseases, especially cancers, have attracted increasing attention (7,8). Alterations or mutations in the lncRNAs have been reported to promote tumorigenesis in multiple cancers, such as prostate, bladder, and kidney cancer (8). According to previous studies, several lncRNAs have been reported to be involved in the progression of CC. lncRNA TUSC8 plays an important role in inhibiting the invasion and migration of CC cells via miR-641/PTEN axis (9). lncRNA MIR205HG acts as a competing endogenous RNA (ceRNA) and modulates tumor growth and progression via sponging miR-122-5p in CC (10). LncRNA HOST2 is demonstrated to be upregulated in HPV-positive CC and promote cell proliferation, migration, and invasion via sponging let-7b (11). lncRNA AB073614 could repress RBM5 to modulate CC cell proliferation and apoptosis (12). Furthermore, lncRNA GHET1 is reported to act as an oncogenic lncRNA in CC (13).

Opa-interacting protein 5 antisense transcript 1 (OIP5-AS1) is a long intergenic noncoding RNA located in human chromosome at 15q15.1 and transcribed in the antisense of OIP5 gene (14–16). Also, OIP5-AS1 has been known to regulate the progression of multiple malignancies, such as breast cancer (17,18), malignant melanoma (19), osteosarcoma (20,21), lung adenocarcinoma (22–24), bladder cancer (25), and colorectal cancer (26). However, the role of OIP5-AS1 in CC is still obscure.

In this study, we clarified a novel function of lncRNA OIP5-AS1 in the regulation of CC progression by inhibition of miR-143-3p to modulate ROCK1 expression. These findings elucidated the significance of lncRNA OIP5-AS1/miR-143-3p interaction in CC, and thus, could further provide a potential therapeutic target for the disease.

## Material and Methods

### Binding sites and mutations

Supplementary Figure S1 shows the binding site of miR-143-3p on OIP5-AS1.

### Protein isolation and western blot

C33As cells were firstly washed with PBS, then lysed in NP-40 buffer for 15 min at 4°C, and isolated for protein extraction. Total protein was quantitated with BCA assay kit (Pierce, USA), followed by electrophoresis using NuPAGE 4–12% Bis-Tris gels (Invitrogen, USA) at 90 V for 1.5 h. Afterwards, we transferred the protein from gel to nitrocellulose membrane using Invitrogen NuPAGE Western transfer system (USA), and the membranes were blocked in 5% milk for 1 h. After the membranes were incubated with primary antibody overnight and HRP-conjugated secondary antibody, the signal was developed with ImageQuant™ LAS 4000 (GE Healthcare Life Sciences, USA).

### RNA extraction and RT-qPCR

Total RNA from objective C33A cells was isolated by TRIzol reagent (Invitrogen), and then quantitated with NanoDrop (Thermo Fisher, USA). The isolated RNA was reverse-transcribed to cDNA for the subsequent qPCR analysis using PrimeScript^®^ RT reagent kit (Takara, Japan) according to manufacturer's protocol. Quantitative RT-PCR was performed in triplicate using an SYBR Premix Ex Taq kit (Takara) in the BioRAD9600 Detection System (Bio-Rad, USA), and GAPDH was used as an internal control.

### Cell proliferation assay

Cell proliferation assay was performed using the Cell Counting Kit-8 assay (CCK-8, Dojindo, Japan) according to the manufacturer's instructions. CC cells were seeded in 96-well plates at a density of 2×10^3^ cells per well. After culture for 0, 24, 48, and 72 h, 10 μL of sterile Cell Counting Kit-8 solution was added to each well followed by incubation for additional 1.5 hours at 37°C. The absorbance values at 450 nm were measured on a Thermo Multiskan MK3 reader (Thermo Fisher Scientific, USA). Six replicate wells were prepared for each experiment group.

### Cell apoptosis analysis

Cell apoptosis assays were carried out with the Annexin V-FITC-PI Apoptosis Detection Kit (Vazyme, Biotech Co., Ltd., China) according to the manufacturer's instructions. C33A cells (1×10^6^ cells per well) were transfected with the indicated plasmids for 48 h, collected with 500 μL binding buffer, and then stained with 5 μL Annexin V-FITC and 5 μL PI. The apoptosis rates were evaluated by flow cytometry.

### Luciferase reporter assay

OIP5-AS1 wild type (WT) and OIP5-AS1 mutant (Mut) sequences were cloned into the pGL3-control vector (Promega, USA) and luciferase reporters were obtained. C33A cells were co-transfected with the reporters, Renilla, and appropriate plasmids using Lipofectamine 3000 (Invitrogen). After post-transfection for about 36∼48 h, cells were harvested and lysed for assay. Cell luciferase activity was detected according to manufacturer's instructions of the Dual-Luciferase^®^ Reporter Assay System (Promega). Renilla luciferase activity was considered as an internal control. All experiments were repeated at least three times.

### RNA immunoprecipitation (RIP)

C33A cells were used to perform RNA immunoprecipitation assay using human anti-argonaute2 (Ago2) antibody (Millipore, USA) and Magna RIP™ RNA-Binding Protein Immunoprecipitation Kit (Millipore) according to the manufacturer's instructions. C33A cell lysates containing lncRNA OIP5-AS1 and miRNA were collected and lysed by RIP buffer, then incubated with magnetic beads conjugated to Ago2 antibody and normal mouse immunoglobulin G (IgG, Millipore). qRT-PCR was used to detect the miRNA in the cell precipitates. All experiments were repeated at least three times.

### Statistical analysis

All data are reported as means±SE, and the significance of the results was determined by unpaired two-tailed Student's *t*-test. Statistical analyses were performed using GraphPad Prism software (USA). P values <0.05 were regarded to be statistically significant.

## Results

### lncRNA OIP5-AS1 was upregulated in CC and was correlated with poor prognosis

**Figure 1:**
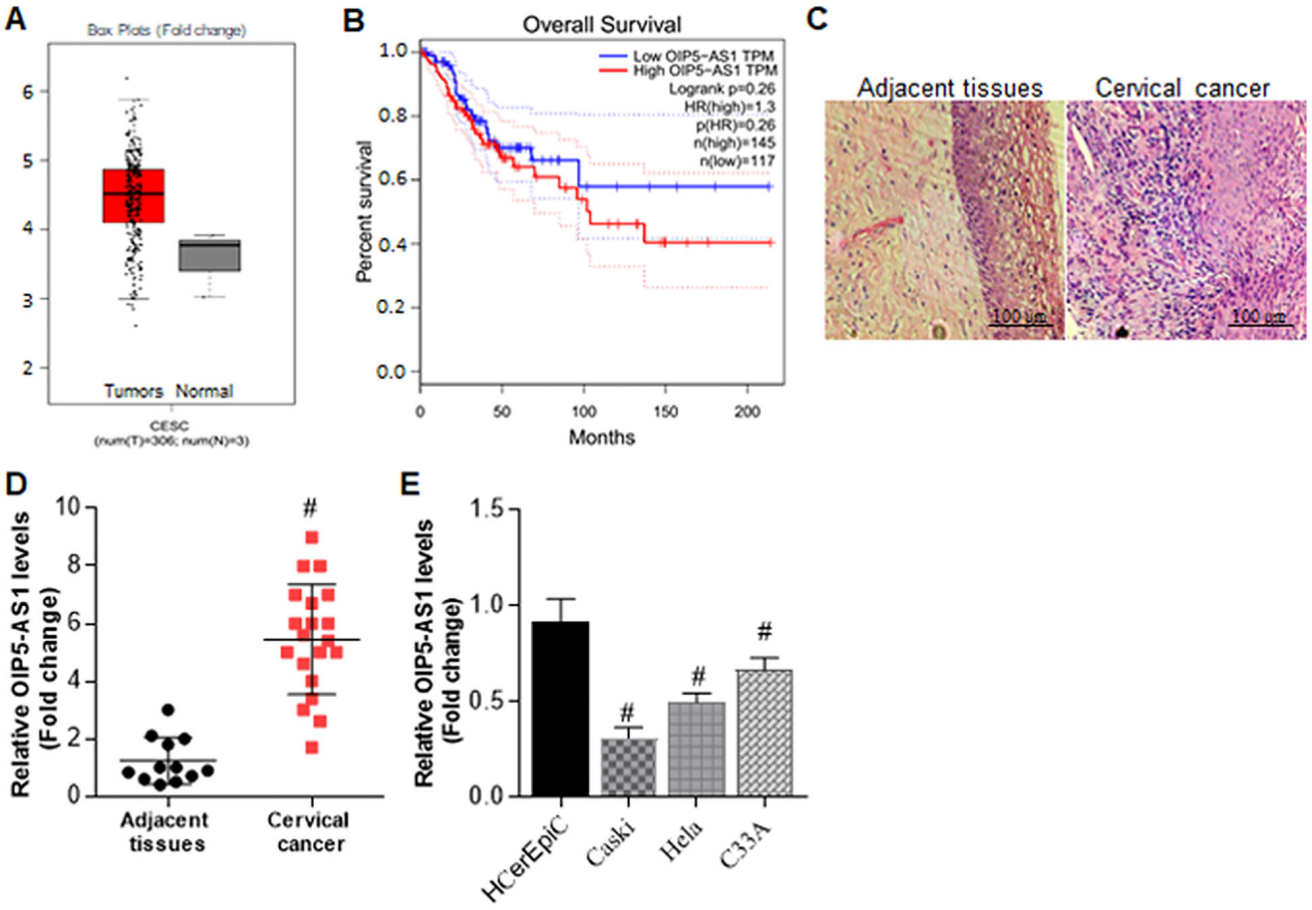
Up-regulation of opa-interacting protein 5 antisense 1 (OIP5-AS1) in cervical cancer patients and its correlation with poor prognosis. **A**, Bioinformatics analysis of OIP5-AS1 expression in cervical cancer tissues and normal tissues according to GEPIA database (median and interquartile range). **B**, Kaplan-Meier survival analysis by OIP5-AS1 levels. **C**, Representative images (400×, scale bar: 100 μm) of hematoxylin-eosin staining in cervical cancer tissues and adjacent normal tissues. **D**, qRT-PCR analysis of OIP5-AS1 expression in cervical cancer tissues and adjacent normal tissues. Data are reported as means±SE (n=20). ^#^P<0.05 *vs* adjacent tissues (Student's *t*-test). **E**, qRT-PCR analysis of OIP5-AS1 expression in different cervical cancer cell lines compared with human cervical epithelial cells (HCerEpiC). Data are reported as means±SE (n=3). ^#^P<0.05 *vs* HCerEpiC controls (Student's *t-*test).

To assess the molecular function of OIP5-AS1 in human CC, we analyzed OIP5-AS1 expression in CC tissues according to GEPIA (Gene Expression Profiling Interactive Analysis, http://gepia.cancer-pku.cn) database by bioinformatics analysis. It was found that OIP5-AS1 expression in CC tissues (n=306, Table 1) was significantly higher than in adjacent normal controls (P<0.05) (Figure 1A). In addition, we examined the overall survival of these patients in terms of OIP5-AS1 expression pattern using the Kaplan-Meier plotter survival analysis. The data showed that the OIP5-AS1 expression level was highly correlated with the outcome (Figure 1B). We then applied hematoxylin-eosin (HE) staining analysis in tumor tissues as well as adjacent normal tissues. As shown in Figure 1C, the cell population in CC tissues was enhanced compared to adjacent normal controls.

**Table 1:** Information of the samples from cervical cancer patients.

Clinicopathological feature	n (%)	OIP5-AS1 (mean±SE)	P value
Age			0.1151
≤50	9 (45)	2.31±0.19	
>50	11 (55)	2.45±0.22	
Menopause			0.1079
Yes	13 (65)	2.19±0.20	
No	7 (25)	2.26±0.24	
Differentiation			0.01083
Well to moderately	14 (70)	2.17±0.25	
Poorly	6 (30)	2.33±0.27	
Grade number (%)			<0.0001
I	3 (15)	1.39±0.21	
II	11 (55)	2.08±0.27	
III	6 (30)	2.64±0.19	
Tumor size			0.0371
<4cm	15 (75)	2.19±0.21	
≥4cm	5 (25)	2.46±0.28	
Lymph node			0.0038
Negative	14 (70)	2.33±0.26	
Positive	6 (30)	2.72±0.15	

OIP5-AS1: opa-interacting protein 5 antisense 1. The unpaired two-tailed Student’s *t*-test was used for statistical analysis.

Furthermore, we found that the expression of OIP5-AS1 was upregulated in CC samples (Figure 1D). We also examined the expression of OIP5-AS1 in different CC cell lines, including Caski, Hela, and C-33 by qRT-PCR analysis. The results showed that OIP5-AS1 expression was significantly elevated in all these cell lines compared with human cervical epithelial cells (HCerEpiC), and C-33 cells showed highest expression of OIP5-AS1 (Figure 1E). Collectively, these results indicated that the elevated expression of OIP5-AS1 in CC patients was associated with poor prognosis, and thus OIP5-AS1 might play an oncogene role in CC progression.

### OIP5-AS1 depletion inhibited cell proliferation and promoted cell apoptosis

**Figure 2:**
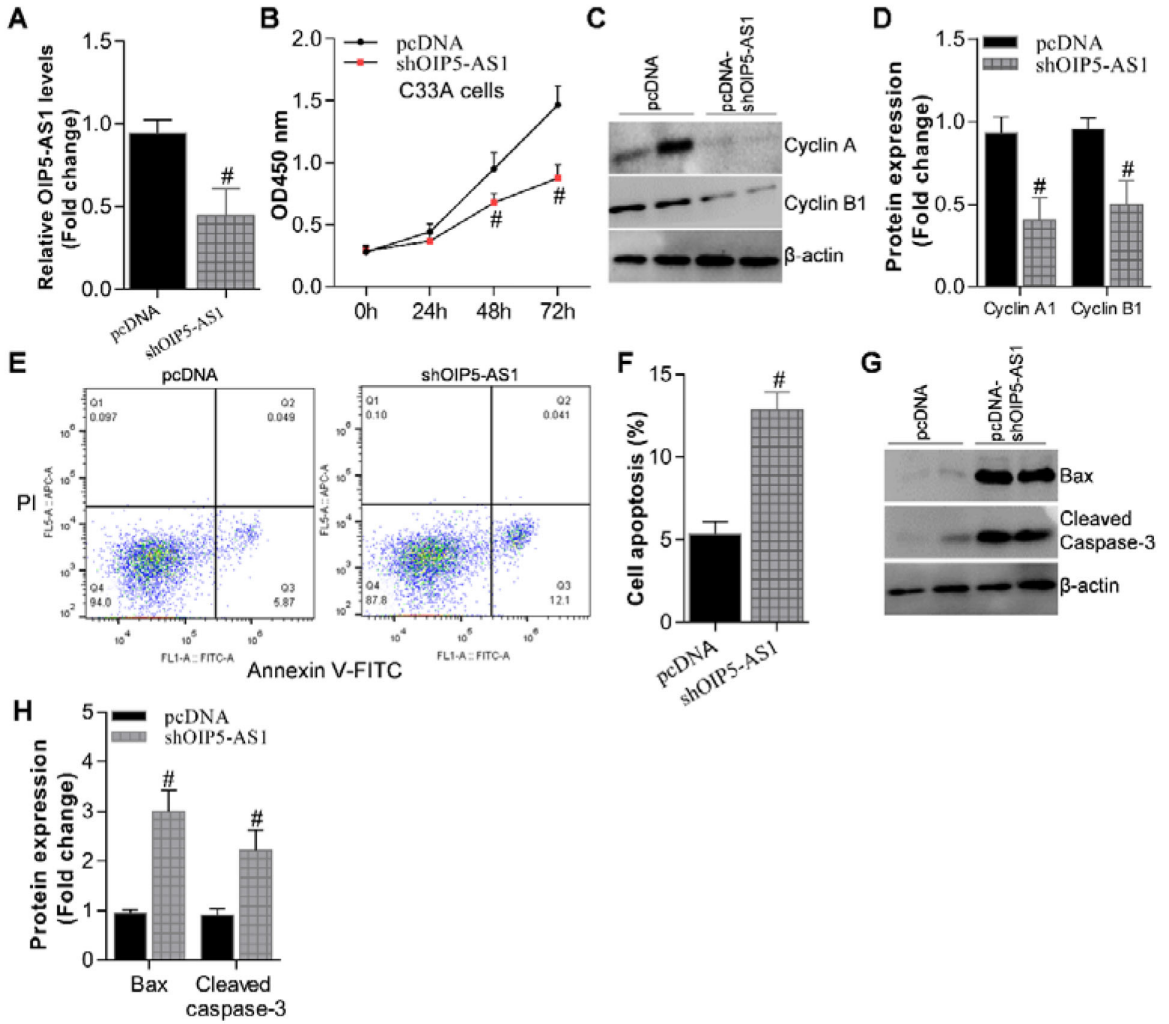
Opa-interacting protein 5 antisense 1 (OIP5-AS1) depletion inhibited cell proliferation and promoted cell apoptosis in C33A cells. **A**, qRT-PCR analysis showed OIP5-AS1 expression was down-regulated in shOIP5-AS1 C33A cells. **B**, CCK-8 cell proliferation assay. **C**, Western blot analysis and (**D**) the quantification of cyclin A and cyclin B1 in OIP5-AS1-depletion C33A cells. **E**, Annexin V-FITC-PI staining in pcDNA C33A cells (control group) and shOIP5-AS1 C33A cells, and **F**, cell apoptosis quantification. **G**, Bax and cleaved caspase-3 expression in OIP5-AS1-depletion C33A cells, and **H,** its fold-change relative to control. Data are reported as means±SE. ^#^P<0.05 *vs* pcDNA controls (Student's *t-*test).

To further investigate the regulation mechanism of OIP5-AS1 on CC, we studied its function by loss-of-function analysis in C-33 cells, because of the highest expression of OIP5-AS1 in this cell line (Figure 1E). We knocked down OIP5-AS1 expression using its specific short hairpin RNA (shRNA), which was verified by qRT-PCR (Table 2) results (Figure 2A). A further CCK-8 proliferation assay demonstrated that OIP5-AS1 depletion dramatically attenuated the cell proliferation potential of C33A cells (P<0.05) (Figure 2B). Consistent with this, western blot analysis of cell cycle markers cyclin A and cyclin B1 also showed their significantly decreased expression in OIP5-AS1-depleted C33A cells compared with normal controls (Figure 2C and D). Moreover, OIP5-AS1 depletion caused cell apoptosis in C33A cells as measured by Annexin V-FITC-PI staining (Figure 2E and F). Besides, we performed western blot assay to check the apoptosis markers Bax and cleaved Caspase-3 expression in these cell population and the results showed their significantly enhanced expression in OIP5-AS1-depleted C33A cells, which was consistent with Annexin V-FITC-PI apoptosis analysis (Figure 2G and H). Together, these data demonstrated that down-regulation of OIP5-AS1 could inhibit cell proliferation and promote cell apoptosis in cervical cancer cells.

**Table 2:** List of the primers used for qRT-PCR.

Primer	Direction	Sequence
hsa-miR-143-3p	forward	5′-TGAGATGAAGCACTG-3′
	reverse	5′-ACTGTACTGGAAGATGGACC-3′
ROCK1	forward	5′-TGAAAGCCGCACTGATGGAT-3′
	reverse	5′-GCCATGAGAAAACACATTGCAG-3′
OIP-AS1	forward	5′-ACCACCGCTGAAACTTCACT-3′
	reverse	5′-AAAATTAGCCAGGCATGGTG-3′

### ROCK1 was a downstream effector of OIP5-AS1 in the regulation of CC

**Figure 3:**
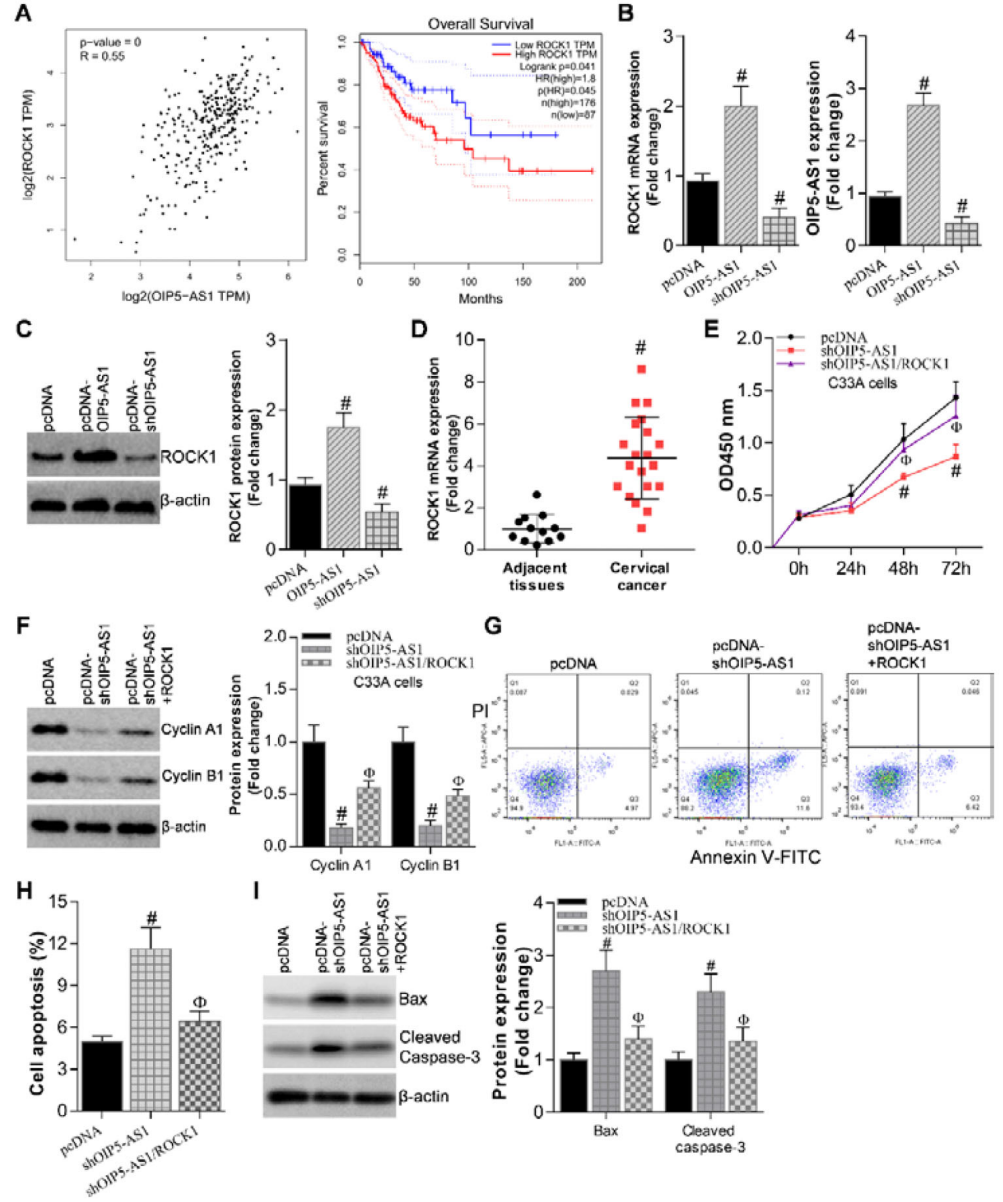
ROCK1 is a downstream effector of opa-interacting protein 5 antisense 1 (OIP5-AS1) in the regulation of cervical cancer. **A**, Bioinformatics analysis showing ROCK1 correlation with OIP5-AS1 (R=0.55) according to GEPIA database (left) and association with poor survival rates of cervical patients (right). **B**, qRT-PCR assay showing OIP5-AS1 overexpression upregulated ROCK1 expression, while OIP5-AS1 depletion downregulated ROCK1 expression in C33A cells. ^#^P<0.05 *vs* pcDNA controls (Student's *t-*test). **C**, Western blot assay showing that OIP5-AS1 positively regulated ROCK1 protein expression. β-actin was used as the internal control. ^#^P<0.05 *vs* pcDNA controls (Student's *t-*test). **D**, qRT-PCR results showing ROCK1 expression in cervical cancer tissues and adjacent normal tissues. ^#^P<0.05 *vs* adjacent tissues (Student's *t-*test). **E**, CCK-8 cell proliferation assay showing that ROCK1 overexpression in OIP5-AS1-depletion cells could partially rescue cell proliferation deficiency. **F**, Western blot assay showing that ROCK1 overexpression rescued cyclin A and cyclin B1 expression in OIP5-AS1-depletion cells. β-actin expression was used as the internal control. **G**, Annexin V-FITC-PI staining, and **H**, data showing that ROCK1 overexpression blocked the aberrant cell apoptosis caused by OIP5-AS1 depletion. **I**, Western blot assay showing that ROCK1 overexpression in OIP5-AS1-depletion cells blocked apoptosis markers Bax and cleaved caspase-3 aberrant expression. β-actin expression was used as the internal control. Data are reported as means±SE. ^#^P<0.05 *vs* pcDNA controls; ^ϕ^P<0.05 *vs* shOIP5-AS1 group (Student's *t-*test).

We then sought to identify the downstream effectors of lncRNA OIP5-AS1 in the regulation of CC. Our bioinformatics analysis according to GEPIA database showed that Rho Associated Coiled-Coil Containing Protein Kinase 1 (ROCK1) expression was positively correlated with OIP5-AS1 (R=0.55; Figure 3A). In addition, as a consequence, high ROCK1 levels were associated with poor survival rates of patients (Figure 3A). qRT-PCR (Table 2) assay verified that OIP5-AS1 overexpression could upregulate ROCK1 expression, while OIP5-AS1 depletion downregulated ROCK1 expression in C33A cells (Figure 3B). Furthermore, western blot assay demonstrated that OIP5-AS1 positively regulated ROCK1 protein expression as well (Figure 3C). Next, as shown in Figure 3D by qRT-PCR, ROCK1 expression was much higher in CC tissues compared with adjacent normal controls (P<0.05).

Moreover, CCK-8 proliferation assay showed that ROCK1 overexpression in OIP5-AS1-depleted cells could partially rescue the cell proliferation deficiency caused by OIP5-AS1 knockdown, indicating that ROCK1 was a functional effector downstream of OIP5-AS1 in CC cells (Figure 3E). Consistently, ROCK1 overexpression could rescue cyclin A and cyclin B1 expression in OIP5-AS1-depleted cells (Figure 3F). On the other hand, ROCK1 overexpression could block the aberrant cell apoptosis caused by OIP5-AS1 depletion, as measured by Annexin V-FITC-PI staining (Figure 3G and H). ROCK1 overexpression in OIP5-AS1-depleted cells could block apoptosis markers Bax and cleaved Caspase-3 aberrant expression (Figure 3I). Taken together, these results demonstrated that ROCK1 was the critical downstream effector of OIP5-AS1 in the regulation of CC progression.

### OIP5-AS1 acted as a molecular sponge of miR-143-3p

**Figure 4:**
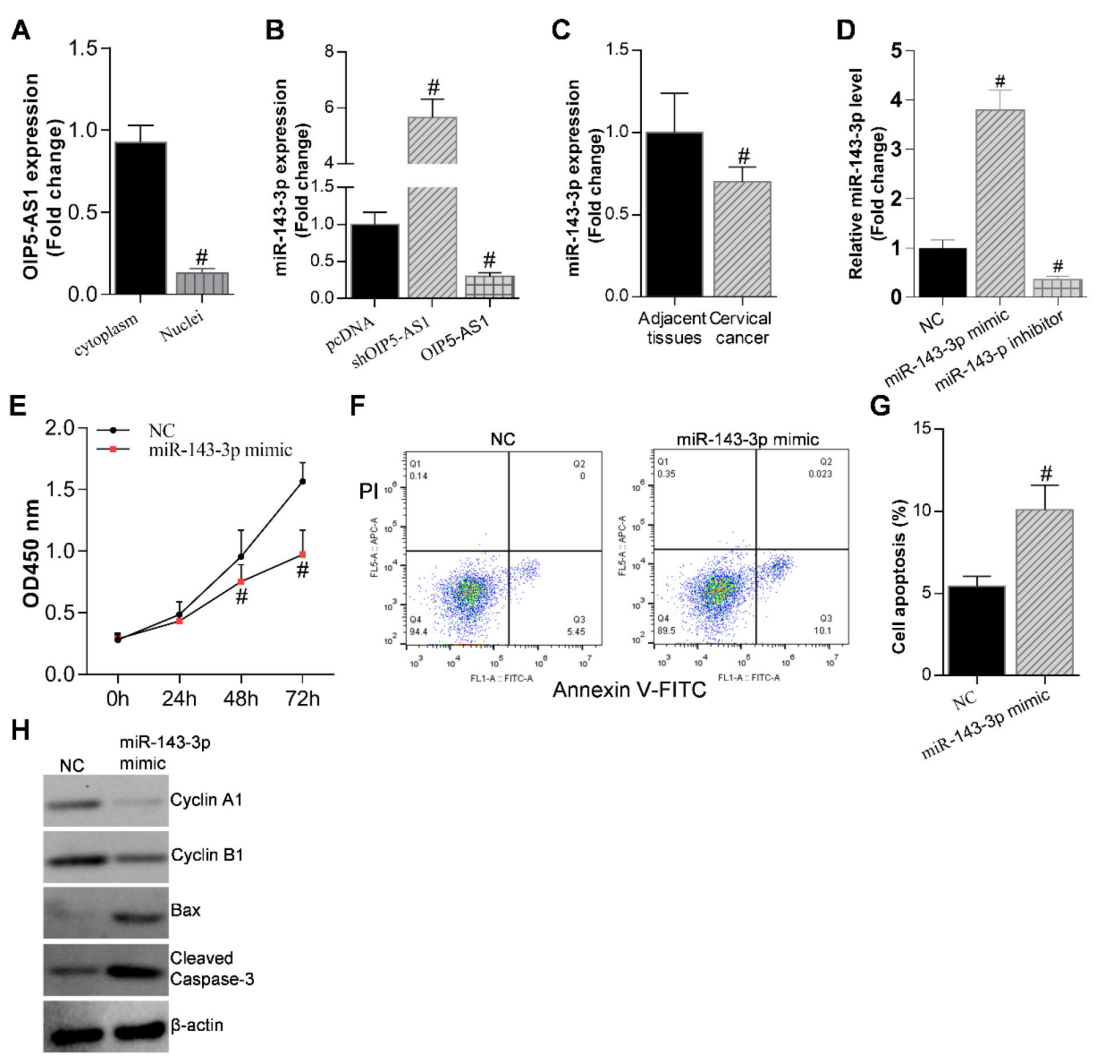
Opa-interacting protein 5 antisense 1 (OIP5-AS1) acted as a molecular sponge of miR-143-3p. **A**, Cytoplasmic and nuclear fractionation assay showed OIP5-AS1 mostly localized in cytoplasm of C33A cells. ^#^P<0.05 *vs* cytoplasm group (Student's *t-*test). **B**, qRT-PCR analysis showing negative correlation between OIP5-AS1 and miR-143-3p. ^#^P<0.05 *vs* pcDNA controls (Student's *t-*test). **C**, qRT-PCR analysis showing miR-143-3p expression in cervical cancer tissues and adjacent normal controls. ^#^P<0.05 *vs* adjacent tissues (Student's *t-*test). **D**, qRT-PCR analysis showing the efficiency of overexpression or knockdown of miR-143-3p in C33A cells. ^#^P<0.05 *vs* negative control (NC) (Student's *t-*test). **E**, CCK-8 cell proliferation assay showing that overexpression of miR-143-3p attenuated C33A cell proliferation. ^#^P<0.05 *vs* NC (Student's *t-*test). **F**, Annexin V-FITC-PI staining, and **G**, corresponding data showing that miR-143-3p overexpression promoted cell apoptosis. ^#^P<0.05 *vs* NC (Student's *t-*test). **H**, Western blot assay showing expression of cyclin A, cyclin B1, Bax, and cleaved caspase-3 in NC cells and miR-143-3p overexpression in C33A cells. β-actin was used as the internal control. Data are reported as means±SE.

lncRNA OIP5-AS1 has been reported to function as a ceRNA and interact with some microRNAs (miRNAs) in multiple cancer cells (27). To determine whether OIP5-AS1 acted as a ceRNA to bind some miRNAs in the regulation of CC, we first checked the localization of OIP5-AS1 in the C33A cells using cytoplasmic and nuclear fractionation assay. It was found that OIP5-AS1 was mostly localized in cytoplasm of C33A cells (Figure 4A). Then, we used TargetScan (http://www.targetscan.org/vert_72/) to predict potential OIP5-AS1-miRNAs interactions, and miR-143-3p was identified. qRT-PCR analysis showed negative correlation between OIP5-AS1 and miR-143-3p (Figure 4B). In OIP5-AS1-depletion C33A cells, the expression of miR-143-3p was enhanced, and vice versa (Figure 4B). In addition, miR-143-3p expression was much lower in cancer tissues compared with adjacent normal controls (P<0.05), as measured by qRT-PCR analysis (Figure 4C) (Table 2). Overexpression of miR-143-3p significantly attenuated C33A cell proliferation by CCK-8 assay (Figure 4D and E). On the other hand, miR-143-3p overexpression promoted cell apoptosis (Figure 4F and G). The expression tendency of cell cycle markers (cyclin A and cyclin B1) and cell apoptosis markers (Bax and cleaved Caspase-3) in miR-143-3p overexpression cells was consistent with the cell phenotypes indicated above (Figure 4H). These data about miR-143-3p molecular function showed negative correlation with that of OIP5-AS1, indicating that OIP5-AS1 acts as a molecular sponge of miR-143-3p in the regulation of CC.

### OIP5-AS1/miR-143-3p interaction regulates ROCK1 expression

**Figure 5:**
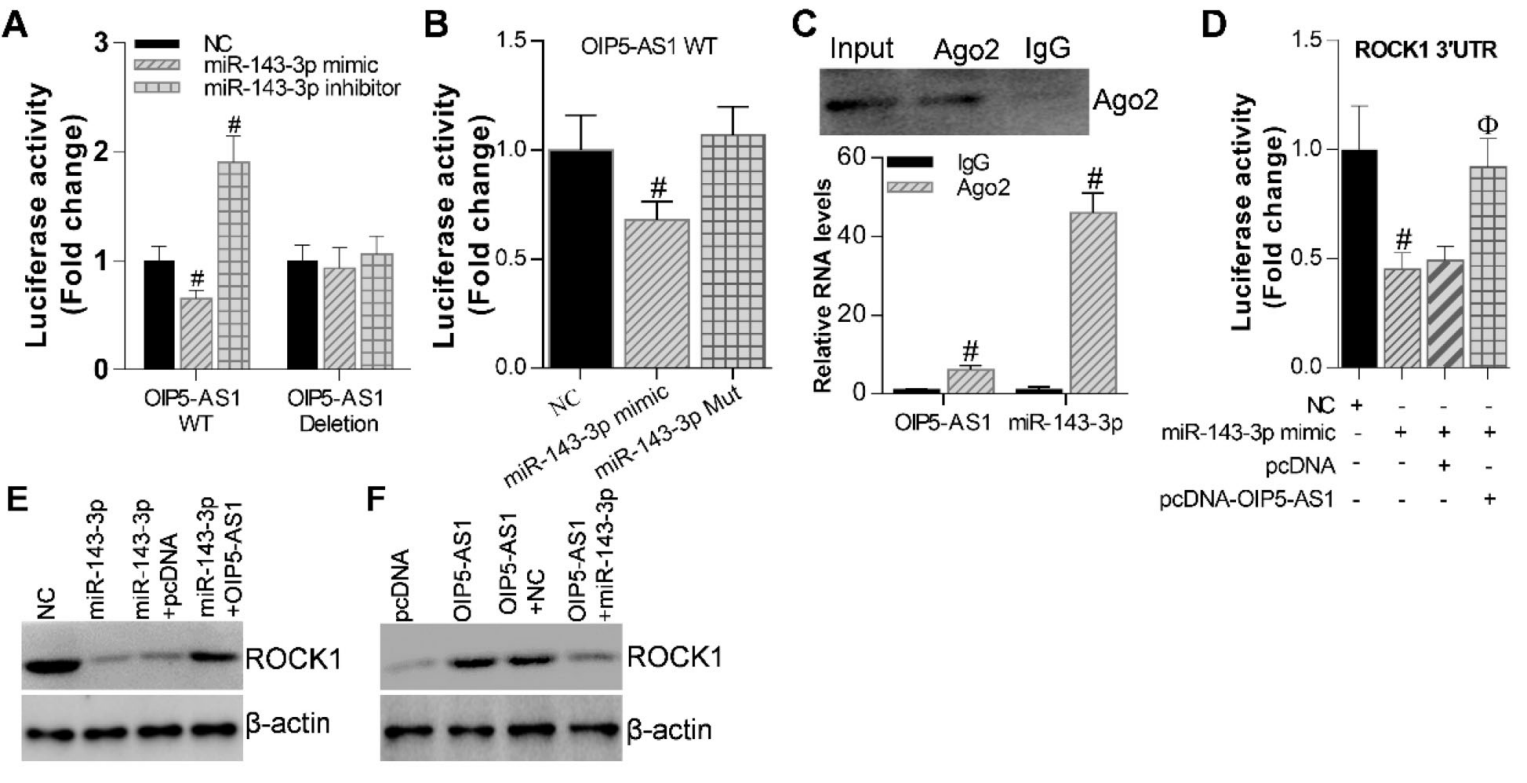
Opa-interacting protein 5 antisense 1 (OIP5-AS1) reversed the inhibitory effects of miR-143-3p in cervical cancer cells. **A**, Relative luciferase in C33A cells transfected with OIP5-AS1-wild type (WT) or OIP5-AS1-Deletion luciferase reporters, accompanied with miR-143-3p mimic or inhibitor. ^#^P<0.05 *vs* NC (Student's *t-*test). **B**, Luciferase assay to test the effect of miR-143-3p mimic or mutant (Mut) on OIP5-AS1 WT-luciferase reporter. ^#^P<0.05 *vs* NC (Student's *t-*test). **C**, RNA immunoprecipitation assay showing that OIP5-AS1 and miR-143-3p could bind with Ago2 protein. ^#^P<0.05 *vs* IgG (Student's *t-*test). **D**, Luciferase assay showing that ectopical expression of miR-143-3p significantly inhibited ROCK1 3′UTR luciferase reporter activity, however, co-expression of OIP5-AS1 could relieve this inhibition. ^#^P<0.05 *vs* NC (Student's *t-*test). ^ϕ^P<0.05 *vs* miR-143-3p+pcDNA group (Student's *t-*test). **E**, Western blot assay showing that overexpression of miR-143-3p decreased ROCK1 expression, while co-expression with OIP5-AS1 could block this depletion. **F**, Western blot assay showing that overexpression of OIP5-AS1 increased ROCK1 expression, while miR-143-3p co-expression relieved this augment. β-actin was used as the internal control. Data are reported as means±SE.

We identified OIP5-AS1 as a molecular sponge of miR-143-3p in CC cells, then we sought to test the binding sites of miR-143-3p in OIP5-AS1. Luciferase reporter assay performed in C33A cells showed that the activity of luciferase reporters containing the theoretical binding sites in lncRNA OIP5-AS1 was inhibited by miR-143-3p overexpression in OIP5-AS1-WT constructs, while miR-143-3p depletion by its inhibitor promoted this reporter activity (Figure 5A). Besides, there was no effect in OIP5-AS1-Mut constructs by miR-143-3p (Figure 5A). Furthermore, overexpression of miR-143-3p binding deficiency mutant form (miR-143-3p Mut), which abolishes its binding with OIP5-AS1, on OIP5-AS1-WT luciferase reporter showed no effect either (Figure 5B). These results demonstrated the binding sites between OIP5-AS1 and miR-143-3p.

It is well known that miRNAs exert their function by binding to anti-Argonaute2 (Ago2), a core component of the RNA-induced silencing complex (RISC) (28). We then performed an RIP assay using Ago2 antibody in C33A cells to test whether lncRNA OIP5-AS1 was associated with an miR-143-3p-component RISC. The results indicated that both OIP5-AS1 and miR-143-3p could bind with Ago2 protein and form an RISC in CC cells (Figure 5C). We then checked the functional interaction between OIP5-AS1 and miR-143-3p to clarify the detailed mechanism of this RISC complex in the regulation of CC progression. Ectopical expression of miR-143-3p could significantly inhibit ROCK1 3′UTR luciferase reporter activity, however, co-expression of OIP5-AS1 could almost relieve this inhibition (Figure 5D). These results suggested that OIP5-AS1 promoted CC cell growth in part by competitively binding miR-143-3p. Western blot analysis demonstrated the antagonism effect between OIP5-AS1 and miR-143-3p in the regulation of ROCK1 expression. Overexpression of miR-143-3p decreased ROCK1 expression, while co-expression with OIP5-AS1 could block this depletion (Figure 5E). On the other hand, overexpression of OIP5-AS1 increased ROCK1 expression, while miR-143-3p co-expression relieved this increase (Figure 5F). Taken together, OIP5-AS1 reversed the inhibition effects of miR-143-3p in CC cells, and OIP5-AS1/miR-143-3p interaction regulated ROCK1 expression.

## Discussion

CC affects millions of women's health worldwide as the fourth most common malignancy (29). However, the pathophysiology of cervical cancer remains little clarified. Only few researchers have reported the connection between lncRNA OIP5-AS1 and CC progression (2,30). Our bioinformatics study demonstrated OIP5-AS1 expression in CC tissues was significantly higher than that in adjacent normal tissues, which is consistent with previous studies (2,30).

We applied multiple biochemistry and cell biology studies to clarify OIP5-AS1 function in cervical cancer, and it was found that OIP5-AS1 depletion inhibited cell proliferation and promoted cell apoptosis, further supporting its oncogene role in cancer progression. As a well-studied key modulator in cancer, ROCK1 exerts its role in cell proliferation, metastasis, and motility. Our study demonstrated ROCK1 was a downstream effector of OIP5-AS1 in the regulation of cervical cancer, and thus the OIP5-AS1-ROCK1 pathway was identified.

MiR-143-3p has been widely studied as a tumor suppressor in several tumors (31,32). Our results demonstrated that OIP5-AS1 promoted cervical cancer cell growth in part by inhibition of miR-143-3p. Furthermore, OIP5-AS1 could reverse the inhibition effects of miR-143-3p in CC cells, and thus, OIP5-AS1/miR-143-3p interaction regulated ROCK1 expression.

OIP5-AS1 has been widely reported to promote tumorigenesis in multiple cancers, including breast cancer, malignant melanoma, lung adenocarcinoma, and colorectal cancer. Our finding about OIP5-AS1 function in CC is consistent with its role in other cancer types, which could further clarify OIP5-AS1 function. To summarize, our study provided deeper understanding of the pathophysiological mechanisms of CC progression, and supported the evidence for the development of therapeutic interventions targeting OIP5-AS1/miR-143-3p-ROCK1 signaling for CC.

## Supplementary material

Click here to view pdf].
